# Protocol for a single-centre randomised pilot study to assess the safety and feasibility of adding a CytoSorb filter during kidney normothermic machine perfusion to remove inflammatory and immune mediators prior to kidney transplantation

**DOI:** 10.1136/bmjopen-2024-093001

**Published:** 2025-03-29

**Authors:** Maithili Mehta, Sarah Hosgood, Michael L Nicholson

**Affiliations:** 1 Department of Surgery, University of Cambridge, Cambridge, UK

**Keywords:** Renal transplantation, Transplant surgery, Transplant medicine

## Abstract

**ABSTRACT:**

**Introduction:**

The introduction of perfusion technologies in kidney transplantation has the potential to improve graft function and survival and increase utilisation. Our previous work demonstrated that kidneys with an enhanced inflammatory and immune response during normothermic machine perfusion (NMP) had significant graft dysfunction after transplantation. The addition of a cytokine filter (CytoSorb) to the NMP circuit dramatically reduces both circulating inflammatory mediators and inflammatory gene expression, but this has not been trialled in clinical practice.

**Methods and analysis:**

This is a randomised phase 1 pilot study to evaluate the safety and feasibility of using a CytoSorb filter in clinical NMP to remove inflammatory and immune mediators. Eligible kidney transplant recipients on the waiting list in the East of England will be approached for consent. A total of 20 patients will be recruited and randomised in a 1:1 ratio for the donor kidney to receive either NMP or NMP with a CytoSorb filter pre-transplantation. The kidney will be transplanted according to standard practice after NMP. The primary endpoint is inflammatory and immune gene expression measured in a cortical biopsy from the kidney 60 min post-transplant. Secondary endpoints include rates and duration of delayed graft function and graft function as assessed by change in creatinine clearance and estimated glomerular filtration rate 2 days, 5 days, 1 month and 3 months post-transplant. Additionally, inflammatory mediators and injury markers will be measured in peripheral blood and urine samples taken pre-operatively and on days 2 and 5 after transplant.

**Ethics and dissemination:**

This study has been approved by the Health Research Authority Health and Care Research Wales Committee (REC 23/WM/0141) and by National Health Service (NHS) Blood and Transplant (Ref: Study 148). Findings will be published in a peer-reviewed journal and disseminated at scientific conferences. The dataset will be made available on request.

**Trial registration:**

The study is prospectively registered on the ISCRTN registry (ID: 13698207).

**STRENGTHS AND LIMITATIONS OF THIS STUDY**

- The study uses a highly sensitive and unbiased measure (inflammatory gene expression in post-reperfusion kidney cortical biopsies) as its primary endpoint.
- The study is not sufficiently powered to support the inference of causal relationships between NMP and clinical outcomes.
- The study is not double-blinded.

## Introduction

 An increasing number of kidneys from extended criteria donors and donation after circulatory death (DCD) donors are being used in transplantation due to the shortage of organs. These marginal kidneys offer a survival benefit to transplant patients compared with remaining on dialysis but portend a higher risk of delayed graft function (DGF) and graft loss compared with alternative kidney allografts.^1 2^ Prolonged DGF is independently associated with longer hospital stays, increased cost, higher incidence of acute rejection, poorer 12-month graft function and higher rates of graft loss and death.^3^,^5^

Traditional hypothermic kidney preservation techniques suppress cellular metabolism and oxygen requirements during transport; however, these conditions cause gradual depletion of energy substrates culminating in cellular damage. After transplantation, restoration of blood flow to the kidney causes further insult via ischaemia reperfusion injury, which involves a cascade of inflammatory and immune mediators that can lead to graft dysfunction.

Normothermic machine perfusion (NMP) offers an alternative organ preservation technique that recirculates an oxygenated red blood cell-based solution through the donor kidney at near physiological pressure and temperature.^6^ It restores oxidative phosphorylation and cellular function following hypothermic preservation and prior to implantation in the recipient. NMP can potentially reduce injury caused by hypothermic conditions and provides a platform for organ assessment and the delivery of targeted treatments. The safety and feasibility of NMP in clinical practice have been established. The results of a randomised controlled trial of NMP in DCD donor kidneys showed no benefit of 1 hour of NMP in reducing rates of DGF post-transplant.^7^ Longer durations of NMP have since been trialled and shown to be safe in clinical practice.^8 9^

Evidence from non-transplanted human kidneys has shown that during NMP, inflammatory and immune mediators are also upregulated similar to the reperfusion response following graft implantation and restoration of blood flow.^10^ Correlations with early outcome after transplantation show that kidneys with an enhanced inflammatory profile after NMP are more likely to have prolonged DGF.^7 10^ Moreover, systemic inflammation early after kidney transplantation is associated with long-term graft loss.^11^

Our group has shown in transplant-declined human kidneys that adding a CytoSorb filter to the kidney NMP circuit removes the inflammatory and immune mediators from the circulating perfusate and attenuates a DGF-associated inflammatory gene signature in renal tissue.^10 12^ CytoSorb is a Class IIb medical device made of a biocompatible and haemocompatible porous bead-based polymer, which adsorbs hydrophobic molecules up to 55 kDa (including cytokines and chemokines) in a concentration-dependent manner.

Clinically, the CytoSorb filter has been used successfully to treat patients with sepsis, COVID-19 and severe inflammatory response syndrome.^13^,^16^ It has also been used in several clinical trials during cardiac bypass.^17^ Experimental studies in lung NMP have shown a beneficial effect after transplantation.^18^

The primary aim of this study is to establish the safety and feasibility of adding a CytoSorb filter in clinical kidney NMP to reduce the inflammatory and immune response within the kidney after transplantation. Secondary outcome measures include rates and duration of DGF and graft function as assessed by change in creatinine clearance and estimated glomerular filtration rate (eGFR) 2 days, 5 days, 1 month and 3 months post-transplant. Additionally, inflammatory mediators and injury markers will be measured in peripheral blood and urine samples taken preoperatively and on days 2 and 5 after transplant.

## Methods

### Study design

**Figure 1:**
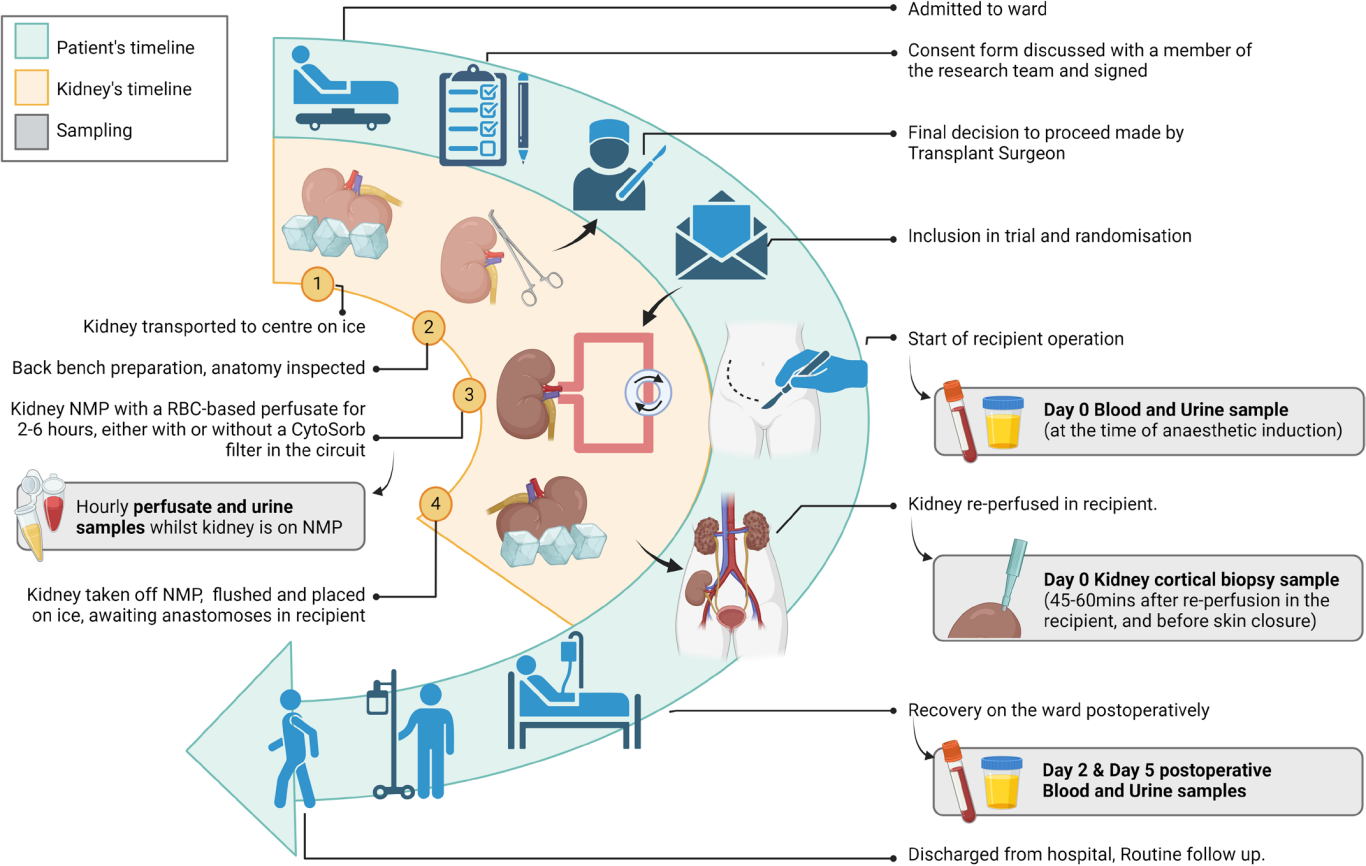
Participant timeline, delineating the patient’s clinical care while enrolled in the trial (blue panel), the kidney’s temporal course (yellow panel) and the key time points for sample collection (grey inserts). NMP, normothermic machine perfusion; RBC, red blood cell.

This is a randomised, single-blinded, single-centre phase 1 pilot study. The protocol is written in accordance with the Standard Protocol Items: Recommendation for Interventional Trials (SPIRIT) guidelines.^19^ Figure 1 shows the study design including the temporal course of events for the patient and the kidney after arrival at the centre and the key time points for sample collection.

### Sample size

A total of 20 patients will be recruited. As this is a pilot study, a formal sample size calculation was not performed. The sample size is pragmatic and based on previous experimental work in human kidneys showing that n=5 per group was able to identify differences in transcriptional changes in human kidneys that were perfused with or without the CytoSorb filter in the perfusion circuit.^10^

### Recruitment

Patients will be recruited from a single centre, Cambridge University Hospitals NHS Foundation Trust (Addenbrooke’s site) between 1 January 2025 and 1 June 2026. The deceased donor kidney transplant waiting list at Cambridge will be used to identify eligible participants. Participant information sheets will be sent to eligible participants beforehand. The transplant co-ordinators will contact the research team when the patient is called into the transplant centre after the identification of a suitably matched donation after brain death (DBD) or DCD donor kidney. Once assessed and the inclusion criteria are met, the patient will be offered the opportunity to ask any further questions.

### Inclusion criteria

Kidney transplant recipients who meet all of the criteria below are eligible to participate.

1. Aged ≥18 years.
2. Either on dialysis (any modality) or predialysis.
3. Undergoing a first or second kidney transplant.
4. Receiving a kidney from a DBD or DCD donor aged ≥50 years.
5. Able to provide written informed consent.

### Exclusion criteria

Kidney transplant recipients who meet any of the criteria below are ineligible to participate.

1. Undergoing a third or subsequent kidney transplant.
2. Receiving a multiorgan transplant (eg, simultaneous pancreas-kidney transplant).
3. Receiving a dual kidney transplant.
4. Receiving a paediatric en bloc kidney transplant.
5. Receiving a kidney from a DBD or DCD donor aged <50 years.
6. Receiving a kidney with complex vascular anatomy.

### Consent

Written informed consent (see online supplemental file) will be obtained by a qualified member of the research team on the day the patient is called into the hospital and prior to the kidney transplant taking place.

### Randomisation

Patients receiving a deceased donor kidney transplant who meet the eligibility criteria and provide informed consent will be randomised in a 1:1 ratio for the donor kidney to receive NMP alone or NMP with the CytoSorb filter prior to transplantation. Randomisation will be performed after the transplant recipient and kidney have both arrived in the transplant centre and a final decision to proceed with transplantation has been made. The randomisation will be performed by a member of the research team using the sealed envelope simple randomisation service. The patient and medical staff caring for the patient after surgery will be blinded to the groups.

### Clinical care

Following organ retrieval, the kidney will be transported to the transplant centre on ice as per standard protocol. Bench preparation of the kidney will take place, and any anatomical abnormalities that preclude inclusion in the trial will be noted.

NMP with or without the CytoSorb filter will then be carried out as described below by members of the research team and clinical staff who are independent of the care of the patient.

The recipient will concurrently be anaesthetised according to local protocols. Antimicrobial prophylaxis, antithrombotic prophylaxis and immunosuppressive medication will be given according to local protocols. It is expected that patients will also receive prophylaxis against *Pneumocystis jirovecii* pneumonia, oral candidiasis and cytomegalovirus.

The kidney will be transplanted using standard techniques into either iliac fossa. The renal artery will be anastomosed to either the common, external or internal iliac arteries and the renal vein to either the common or the external iliac vein. The ureteric anastomosis will be performed as an extravesical onlay over a double J stent.

A cortical 4 mm punch biopsy of the kidney will be taken postreperfusion (45–60 min), and recipient blood and urine samples will be collected at predetermined time points as described below.

### Study groups

#### NMP

Kidneys will be placed on the NMP system (Kidney Assist, XVIVO) and perfused with a red blood cell-based solution mixed with a priming solution (human serum albumin 5%, Ringer’s solution, dexamethasone, heparin, meropenem, calcium gluconate 10% and sodium bicarbonate 8.4% to adjust pH to be within the normal range). Infusion pumps will deliver the following:

- 5% glucose 2–4 mL/hour as required.
- Amino acid/electrolyte solution (with 100 units of Actrapid insulin, 15 mL sodium bicarbonate 8.4% and 5 mL Cernevit) 5–10 mL/hour.
- Prostacyclin 0.5 mg (in 100 mL 0.9% sodium chloride) 3 mL/hour as required.
- Verapamil 2.5 mg as required.
- Ringer’s lactate solution to replace urine output.

Kidneys will be perfused for a minimum of 2 hours and a maximum of 6 hours at a mean arterial pressure of 75–85 mmHg and temperature of 35–37.4°C. Renal blood flow (RBF) will be monitored continuously during NMP. Intrarenal resistance (IRR) will be calculated (mean arterial pressure/RBF) until the end of perfusion. Blood gas analysis will be used to measure the acid-base balance during NMP.

After NMP, kidneys will be flushed with cold preservation solution and placed on ice until transplantation.

#### NMP with CytoSorb filter

**Figure 2:**
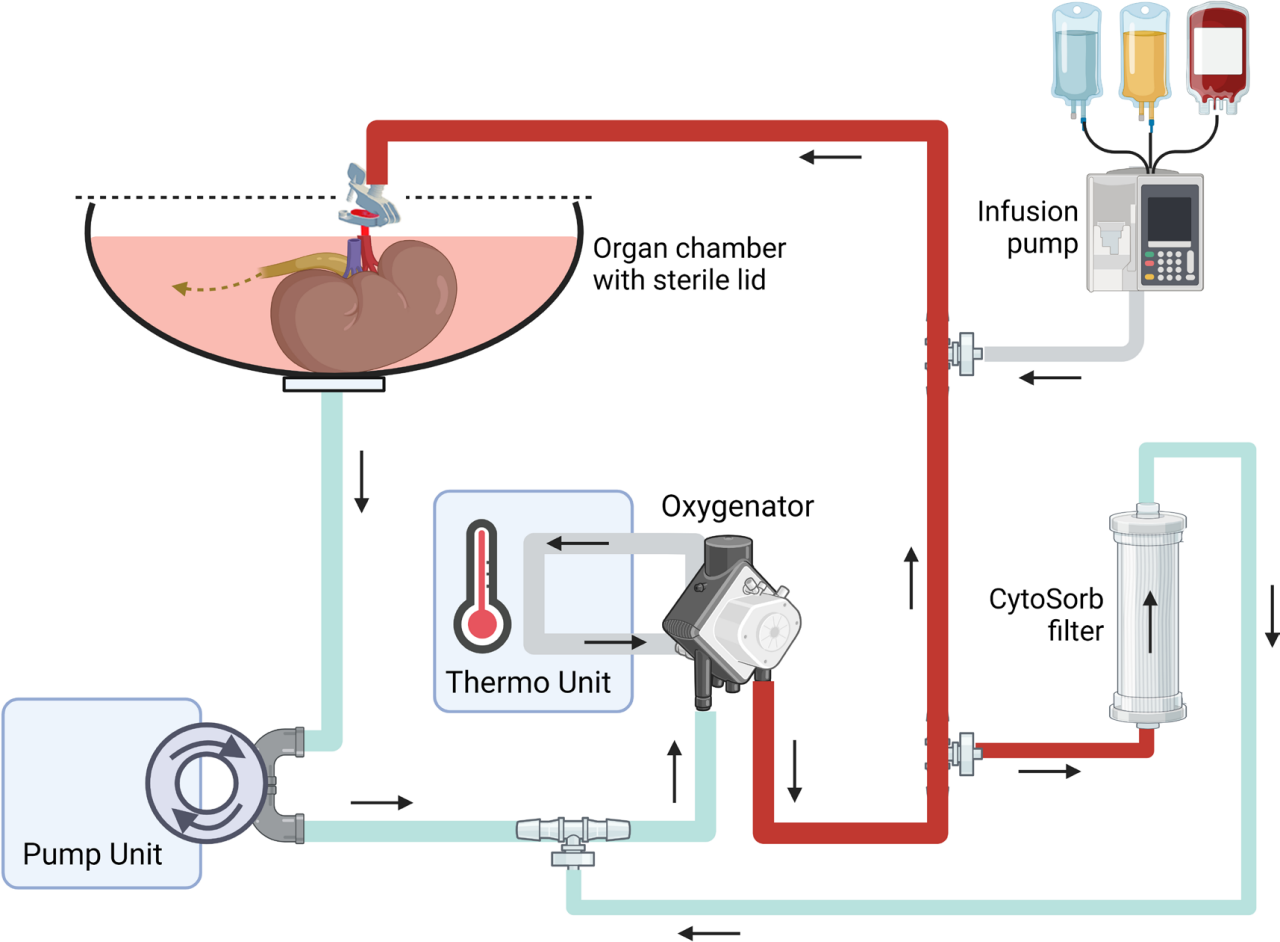
Schematic representation of the XVIVO Kidney Assist perfusion circuit with a CytoSorb filter incorporated. The kidney is placed in a sterile organ chamber with a lid. A patch clamp is used to connect the arterial end of the tubing to the renal artery. The renal vein and ureter drain directly into the venous reservoir (urine recirculation). Additional component parts (not shown) include a pressure transducer, an arterial flow sensor, temperature sensors and sampling ports.

Kidneys will be perfused as per the standard NMP protocol but with the CytoSorb filter (CytoSorbents Europe GmbH) added to the circuit for the duration of perfusion (figure 2).

### Follow-up

Patients will be followed up at the transplant clinic or local centre at 1 and 3 months as normal practice after discharge from the hospital.

### Withdrawal from the study

Withdrawal from the study is likely to be uncommon but may occur on account of withdrawal of consent by the patient or the kidney being deemed untransplantable following final bench surgery.

Patients who are randomised but withdraw before intervention will receive standard clinical care according to the local protocol. If patients undergo the intervention but subsequently withdraw, they will also receive standard clinical care. In the unexpected situation where consent to use data and samples that have already been collected is withdrawn, these will be discarded.

### Protocol deviations

One-off protocol deviations will be documented and reported to the chief investigator and sponsor. Frequent deviations will be reported to the sponsor and Research Ethics Committee.

### Patient and public involvement

This research proposal was presented to the NHIR Blood and Transplant Research Unit in Organ Donation and Transplantation (BTRU in ODT) Patient and Public Research Panel who provided guidance on the protocol design. On completion of the study, the results will be presented to panel members at an annual NIHR BTRU in ODT progress meeting.

### Outcomes

#### Primary outcome

The primary outcome measure is inflammatory and immune gene expression measured in postreperfusion renal cortical biopsies.

#### Secondary outcomes

The secondary outcome measures are:

1. Rates of DGF, defined as the need for dialysis within the first 7 postoperative days.
2. Duration of DGF.
3. Incidence of primary non-function (PNF), defined as dialysis dependence or creatinine clearance ≤20 mL/min at 3 months post-transplant.
4. Graft function at 3 months post-transplant as measured by eGFR calculated using the Chronic Kidney Disease Epidemiology Collaboration (CKD-EPI) equation.
5. Graft survival at 3 months post-transplant.
6. Patient survival at 3 months post-transplant.
7. Incidence of biopsy-proven acute rejection.
8. Complications within 3 months of transplant (infection, re-operation due to bleeding).
9. Length of hospital stay.
10. Levels of inflammatory/immune and injury markers in perfusate and urine samples taken during NMP.
11. Isolation of peripheral blood mononuclear cells from recipient blood samples pretransplant and on days 2 and 5 post-transplant to examine the inflammatory/immune response.
12. Biomarkers of kidney injury on days 2 and 5 post-transplant.

### Samples and analysis

#### Sample collection

- A cortical 4 mm punch biopsy of the kidney will be taken from the transplant kidney at 45–60 min after reperfusion. Tissue samples will be divided. Half will be fixed in 10% formalin and the other half placed in RNA later.
- Samples of the perfusate and urine (if any) from the kidneys during NMP will be collected at the start and hourly until the end of NMP.
- Peripheral blood (10 mL) and urine (10 mL) samples from the transplant recipient will be collected at the start of surgery and then at days 2 and 5 post-transplant.

#### Sample storage

Samples will be processed as required and then stored at –80°C or in liquid nitrogen. Frozen tissue samples will be stored in the research laboratories at the University of Cambridge, department of surgery. Fixed tissue will be processed by the tissue bank at Addenbrooke’s Hospital and stored within the department of surgery laboratories.

Biological samples collected from participants as part of this trial will be transported, stored, accessed and processed in accordance with national legislation and the requirements set out in the 2004 Human Tissue Act. Samples will be labelled in compliance with the 1998 Data Protection Act. On completion of the trial, samples will be disposed of in accordance with the Human Tissue Authority’s Code of Practice.

#### Data collection and storage

Patient-related data will be collected from computerised health records. Each participant will be allocated a study code to facilitate data anonymisation for storage on a password-protected database.

#### Analysis

- Donor and recipient characteristics, levels of creatinine clearance, eGFR, graft and patient survival, complications, length of hospital stay, incidences of DGF and PNF, and episodes of acute rejection and perfusion parameters during NMP will be compared between the two groups using the appropriate statistical tests.
- Cortical kidney tissue will be analysed as follows: RNA will be extracted from RNAlater biopsies and bulk sequenced. The data will thereafter be demultiplexed, assessed for quality control using FASTQC, the Fastq files aligned to the human genome using Hisat2 and normalisation and differential gene expression analysis performed using DESeq2. RNA extraction, library preparation, sequencing and sample analysis will be performed at a laboratory with expertise in this area. Additionally, fixed tissue will be used to assess the level of injury and inflammation.
- Samples of perfusate and urine from the kidney during NMP, and samples of urine from the recipient will be analysed as follows: levels of cytokines will be measured using a Cytokine Luminex panel. Biomarkers will be quantified using a Proteome Profiler Human Kidney Biomarker Array Kit. Changes in the level of cytokines and injury markers will be calculated and compared between groups using repeated measures and analysis of variance.
- Peripheral blood samples from recipients will be analysed as follows: peripheral blood mononuclear cells will be isolated from whole blood using density-gradient separation, and their relative abundance, phenotype and activation status will be determined using cell sorting, flow cytometry, transcriptomic analysis and cell culture techniques. Levels of inflammation/immune mediators will be measured using a Luminex panel.

Cytokines of particular interest are Granulocyte colony-stimulating factor, Granulocyte-macrophage colony-stimulating factor, Interferons alpha and gamma, Interleukins (IL)-1beta, 1 receptor antagonist, -2, -4, -5, -6, -7, -8, -10, -12, -13, -15, -17, and -18, and Tumour necrosis factor alpha (TNF-α). Chemokines of interest are CXCL2, CXCL3, CXCL4 and CCL5. Biomarkers of interest include C-reactive protein, Prostaglandin E2, Prostacyclin, Thromboxane B1, High mobility group box-1 protein, Beta-2 microglobulin, Neutrophil gelatinase-associated lipocalin, and Kidney injury molecule-1 among others.

Analysis of the RNAseq data will focus particularly on the expression of TNF-α signalling via Nuclear factor kappa B, Mammalian target of rapamycin complex-1 signalling, inflammatory response, P53 pathway, IL-2 signalling and Transforming growth factor beta signalling as these were the most upregulated genes in our previous study^10^.

## Discussion

### Anticipated benefits

To our knowledge, this is the first clinical study to test the safety and feasibility of using CytoSorb therapy during kidney NMP.

It is anticipated that the analysis will provide novel mechanistic insights. The use of global transcriptomic techniques on postreperfusion kidney biopsies will elucidate the role played by cytokine signalling in reperfusion injury to the graft. Further, the isolation and characterisation of peripheral blood mononuclear cells and inflammatory mediators from recipient blood samples taken in the days following transplant will provide valuable information about the host systemic inflammatory response in the early postoperative period and whether this is altered by pretransplant CytoSorb therapy.

While not its primary aim, the study will contribute to the evidence base for the safety and feasibility of intermediate durations of kidney NMP (2–6 hours).

### Limitations

The study design has some limitations. First, while the sample size is large enough to prove feasibility, the study is not sufficiently powered to draw causal inferences about the relationship between the interventions and clinical endpoints. However, the results will inform future efficacy trials. Second, randomisation does not account for donor type; therefore, there is a small risk of imbalance in the number of DBD/DCD donor kidneys between the two study arms.

Finally, a clinical and ethical decision was made to take a single renal cortical biopsy postreperfusion to minimise the risk of bleeding. The lack of a pretransplant (pre-NMP) biopsy makes it difficult to assess the contribution of baseline inflammation in the donor allograft at the time of organ retrieval, as well as the impact of NMP on the inflammatory status of the donor kidney. To mitigate the impact of this on the analysis, access to Quality in Organ Donation National Biobank renal cortical biopsy samples taken at the time of organ retrieval will be requested retrospectively, where available.

### Ethics and dissemination

#### Ethics

This study has been approved by the Health Research Authority Health and Care Research Wales Committee (REC 23/WM/0141) and by NHS Blood and Transplant (Ref: Study 148).

#### Confidentiality

Patient identifiable information will be accessed and handled by members of the research team in compliance with the 1998 Data Protection Act. Only patient unidentifiable data will be transmitted to the sponsor and co-investigators. The data will be stored for 5 years after the end of the study. The custodian of the data will be the chief investigator.

#### Adverse events

The risk to participants entering the study is low. Any adverse events directly linked to the intervention will be recorded and reported to the chief investigator and sponsor immediately.

#### Indemnity

Cambridge University Hospitals NHS Foundation Trust will accept full financial liability for harm caused to participants in the study through the negligence of its employees and honorary contract holders. The University of Cambridge will arrange insurance for negligent harm on account of protocol design and for non-negligent harm arising through participation in the study.

#### Dissemination

Findings will be published in a peer-reviewed journal and disseminated at scientific conferences. The dataset will be made available on request.

## supplementary material

10.1136/bmjopen-2024-093001 online supplemental file 1
