# Supplementary material for: Protocol for a single-centre randomised pilot study to assess the safety and feasibility of adding a CytoSorb filter during kidney normothermic machine perfusion to remove inflammatory and immune mediators prior to kidney transplantation
Source: BMJ Open. 2025 Mar 29;15(3):e093001. doi: 10.1136/bmjopen-2024-093001 (PMC11956346; doi:10.1136/bmjopen-2024-093001)
Supplement: online supplemental file 1 [file bmjopen-15-3-s001.docx]

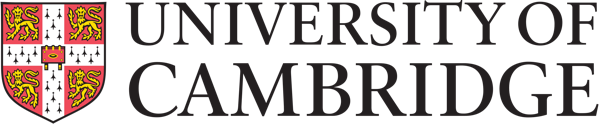


**Participant Information Sheet**

**IRAS:** 322728

**Sponsor:** Cambridge University Hospitals NHS Foundation Trust and The University of Cambridge

**Study title**

A randomised pilot study to assess the safety and feasibility of adding a Cytosorb filter during kidney normothermic machine perfusion.

**Invitation and brief summary**

You are being invited to take part in a research study. Before you decide, it is important for you to understand why the research is being done and what it will involve. Please take time to read the following information carefully and discuss it with others if you wish. Ask us if there is anything that is not clear or if you would like more information. Take time to decide whether or not you wish to take part.

In this research study, we would like to assess whether a new technique of kidney preservation is beneficial.

**What is involved?**

When kidneys are removed from an organ donor they are normally stored on ice until they are ready to be transplanted. A kidney can be preserved safely at a low temperature in these conditions. However, there is some degree of deterioration and the longer they are left in this condition the more they deteriorate (rather like food that is kept in the fridge).

We have developed a technique that may improve the quality of the kidney. This involves placing the kidney on a machine and passing a warmed, oxygen-rich solution containing red blood cells through it. Under these conditions the kidney can start to function again and produce urine. We have trialled this in patients with no adverse effects. From our research we know that whilst being perfused, kidneys release cells that cause inflammation. In a small number of patients, we want to test whether adding a specialised filter to the machine has any beneficial effects.

**What would taking part involve?**

You will be prepared for surgery in the normal way. Standard practice involves keeping the transplant kidney under cold storage in ice until the time of the transplant operation. If you consent to take part in this trial then your kidney will be randomly assigned to having your kidney placed on a machine and warmed with an oxygenated blood-based solution. In one group a special filter will be added to the circuit to reduce the risk of inflammation. Should there be any problem with the warm perfusion procedure then the kidney can be quickly removed from the perfusion machine and returned to cold storage in ice before transplantation.

During the transplant operation we will also take a small tissue biopsy from the kidney before after transplantation. Although there is a small risk of causing bleeding from the kidney biopsy site (<5%) your surgeon will be able to repair the bleeding site if this happens.

After your transplant you will receive the normal standard care but will also be asked to provide a few additional blood and urine samples for analysis. Your participation in this study will not affect the way you are followed up after a transplant. The normal follow up involves clinical visits at least twice a week for six weeks and then weekly for a further six weeks.

The data collected will be stored on a secure database and tissue, blood and urine samples within secured laboratories only accessed by the transplant research team. Once the samples have been analysed, they will be disposed as per HTA guidance.

**What are the possible benefits of taking part?**

This study is being performed to test whether the specialised filter added during warm perfusion can improve the condition of the kidney. The first step is to assess whether this is practical before carrying out a larger trial. We cannot guarantee that this will improve the outcome of your kidney transplant but it will help us to improve future techniques of kidney preservation. This may enable us to transplant more kidneys in the future.

**What are the possible disadvantages and risks of taking part?**

There are no potential side effects to you. This technique of warm perfusion is applied to the kidney only, before it is transplanted. There is a small risk that the kidney might be damaged during the assessment and therefore could not be transplanted. This has not happened in our experience of 200 cases so far but it remains as a potential risk.

**What happens when the research study stops?**

At the end of the research study, you will continue to be followed up for your kidney transplant either at Addenbrooke’s Hospital or at your local renal hospital.

**What if new information becomes available?**

If new information becomes available your Transplant Consultant might consider it to be in your best interests to withdraw you from the study. He/she will explain the reasons and arrange for your care to continue.

**What will happen if I do not want to carry on with the study?**

You will be given normal care after the kidney transplant. If you withdraw from the study, we will ask permission to use the data collected up to your withdrawal.

**What will happen to the results of the research study?**

The results of the research will be published in specialist journals in order to inform other transplant doctors around the world. You will not be identified in any report or publication. You will be able to get a copy of the results by asking the kidney doctors in the follow up clinic.

**Will my General Practitioner/Family Doctor (GP) be involved?**

Participation in this trial will not affect your treatment and follow-up by your GP after discharge from the hospital.

**Who is organising the research?**

The research is being organised by the Transplant Research Team, Department of Surgery, University of Cambridge and at the Cambridge Transplant Unit

**Who has reviewed the study?**

The study has been reviewed by CUH research advisory committee and the Local Research Ethics Committee.

**Will my taking part in this study be kept confidential?**

We will follow ethical and legal best practice and all information about you will be handled confidentially. If you join the study, some parts of your medical records and the data collected for the study will be looked at by authorised persons from the research team. They may also be looked at by representatives of the regulatory authority or by those responsible for research and development audit (for monitoring the quality of the research). All have a duty of confidentiality to you as a research participant and will do their best to meet this duty. Our procedure for handling, processing, storage and destruction of data will match the *Data Protection Act 1998*. Your name will not be disclosed outside the hospital. The data collected will be stored and retained securely for 10 years and it will also be disposed of securely. You have the right to check the accuracy of data held about you and correct any errors.

**What if there is a problem?**

Any complaint about the way you have been dealt with during the trial or any possible harm you might suffer will be addressed. If you have any concerns about any aspect of this trial you should speak to your trial doctor who will do their best to answer your questions.

In the event that something does go wrong and you are harmed by taking part in the research and this is due to someone’s negligence then you may have grounds for a legal action for compensation against Cambridge University Hospitals NHS Foundation Trust or the University of Cambridge. The normal National Health Service complaints mechanisms will still be available to you (if appropriate). The University has obtained insurance, which provides no-fault compensation i.e. for non-negligent harm, you may be entitled to make a claim for this.

**Obtaining further information**

If you have any questions or concerns about any aspect of this study, you should ask to speak to the researchers who will do their best to answer your questions or to Professor Michael Nicholson (01223 339221)/ Dr Sarah Hosgood (01223 763105)

**Complaints and Independent Advice**

If you wish to speak to an independent body about any concerns or complaints about any aspect of the way you have been approached or treated during this trial, you can do this through the Addenbrooke’s Kidney Patient’s Association or the Patient Advice and Liaison Service (PALS) at Addenbrooke’s Hospital. The formal NHS complaints procedure is also available to you. Details can be obtained through the hospital.

**Complaints**

If you remain unhappy and wish to complain formally, you can do this through the NHS complaints procedure.

**NHS based research**

If you are harmed by taking part in this research project, there are no special compensation arrangements. If you are harmed due to someone’s negligence, then you may have grounds for a legal action but you may have to pay for it. Regardless of this, if you wish to complain, or have any concerns about any aspect of the way you have been approached or treated during the course of this study, the normal National Health Service complaints mechanisms will be available to you.

**Contacts for further information**

A) General information about research can be found on [www.nres.org.uk](http://www.nres.org.uk); [www.addenbrookes.nhs.uk](http://www.addenbrookes.nhs.uk); or [www.instituteofclinicalresearch.org.uk](http://www.instituteofclinicalresearch.org.uk)

B) For specific information about this research project, contact Professor Michael Nicholson, 01223 339221.
