# Supplementary material for: Protocol for a single-centre randomised pilot study to assess the safety and feasibility of adding a CytoSorb filter during kidney normothermic machine perfusion to remove inflammatory and immune mediators prior to kidney transplantation
Source: BMJ Open. 2025 Mar 29;15(3):e093001. doi: 10.1136/bmjopen-2024-093001 (PMC11956346; doi:10.1136/bmjopen-2024-093001)
Supplement: Supplementary file 2 [file bmjopen-2024-093001.reviewer_comments.pdf]

PEER REVIEW HISTORY

BMJ Open publishes all reviews undertaken for accepted manuscripts. Reviewers are asked to complete a checklist review form and are provided with free text boxes to elaborate on their assessment. These free text comments are reproduced below.

ARTICLE DETAILS

Title (Provisional)

Protocol for a single centre randomised pilot study to assess the safety and feasibility of adding a CytoSorb® filter during kidney normothermic machine perfusion to remove inflammatory and immune mediators prior to kidney transplantation

Authors

Mehta, Maithili; Hosgood, Sarah; Nicholson, Michael L

VERSION 1 - REVIEW

**Reviewer**	**1**
**Name**	**Ho, Quan Yao**
**Affiliation**	**Singapore General Hospital**
**Date**	**04-Nov-2024**
**COI**	**None**

I thank the editors and authors for the opportunity to review this protocol.

The authors presented a protocol for a phase 1 RCT to test the addition of the CytoSorb filter to a NMP circuit for deceased donor kidney transplants.

This study will help to answer an important clinical question. The protocol is clear and well written and I have only minor comments.

- Are there any additional treatment required for the graft while it is connected to the Cytosorb filter while on pump (e.g. additional anticoagulation)?

- Will the hemodynamic parameters of the graft while on NMP be monitored?

- What are the device-specific adverse events that will be monitored for during the study?

- Can inflammatory markers be measured from blood samples from the deceased donors?

- Can the authors further elaborate on the inflammatory/injury markers and markers of kidney injury that they plan to measure / are available in the panels that will be used?

- Can the authors provide further details on the gene expression analysis since it is a primary aim (e.g. platform and specific analysis)?

- Was a sample size calculation performed for the primary aim proposed?

- It may be interesting to monitor perioperative hypotensive episodes and/or use of vasopressors

---

**Reviewer**	**2**
**Name**	**Knight, Simon**
**Affiliation**	**University of Oxford**
**Date**	**04-Dec-2024**
**COI**	**None**

This is an interesting study and a well written, clear manuscript. The primary focus is safety and feasibility, rather than efficacy.

My main suggestions relate to a lack of justification or details:

1. Inclusion criteria are faily restrictive for a single centre study (only donors > 50, recipients on dialysis). Could the authors justify these decisions? For example, as the main focus is safety and feasibility, is there a reason to exclude pre-dialysis recipients?

2. There is no stratification by donor type in randomisation. Whilst I suspect this is due to the small sample size, there is a risk in such a small study of an imbalance in the DBD/DCD ratio between arms which would significantly affect the primary outcome. This should at lease be noted as a potential risk.

3. Is there a maximum perfusion duration specified in the protocol?

4. As the perfusionists will not be blinded, what steps have been taken to standardise the perfusions? A number of drugs can be infused "as required" leaving the potential for manipulation of the perfusion, for example? Does the study protocol standardise the use of such infusions (e.g. if resistance > x, give more verapamil or similar)?

5. Whilst withdrawals are unlikely, will these be replaced in the randomisation schedule (e.g. inf the kdiney is not transplanted)? As the sample size is small, one or two withdrawals could have a significant impact.

6. Does the addition of the cytokine filter constitute a modification o the disposable circuit? Or are the device and circuit considered to be used "on-label" (i.e. does this need to be registered as a device trial with the MHRA)?

7. The outcomes need to be defined in much more detail. What inflammatory and immune gene markers will be analysed? These should be pre-specifies to avoid "fishing". What definitions of DGF and PNF will be used? Howe will transplant function be assessed? etc.

8. The study is described as a safety and feasibility study, yet the primary endpoint is an efficacy endpoint. How did the authors arrive at the proposed sample size? Purely a feasible recruitment target, or is there any experimental data to demosntrate a likely difference in the primary endpoint?

9. The authors refer to QUOD in the discussion. This should be defined for a non-transplant audience. What proportion of the donors do the authors anticipate having QUOD samples available for?

10. Figure 1 is an excellent summary of the study. The only step missing is the provision of information to patients on the WL (very minor, not essential!).

**Reviewer**	**3**
**Name**	**Bishop , Nicolette**
**Affiliation**	**Loughborough University**
**Date**	**10-Dec-2024**
**COI**	**None**

This is a well designed study with high potential importance. The protocol has been reported in line with the SPIRIT guidelines and as such my comments are minor and only for clarification.

1) the dates for the study should be included somewhere in the manuscript, in line with the editorial requirements for protocol papers in BMJ Open.

2) Analysis-page 9: While it is acknowledged that the methods will assess panels that include many different genes, cytokines and other biomarkers, it would be useful to highlight some key example expressed genes, cytokines, immune phenotypes and other biomarkers that will be assessed here, particularly for interpretation of the benefits of the future results to elucidate specific relevant mechanisms.

**Reviewer**	**4**
**Name**	**Hunter, James**
**Affiliation**	**University Hospitals Coventry and Warwickshire NHS Trust**
**Date**	**13-Dec-2024**
**COI**	**None**

Thank you for the opportunity to review this interesting early phase clinical trial protocol. The study aims to randomise kidneys (n=20) to NMP with and without a Cytosorb filter to determine the role on inflammation and inflammatory gene expression.

The aims are clear and limitations appropriately described.

minor comments

NMP duration of 2-6h - presumably the differences in inflammation and gene expression will vary depending upon the NMP duration. I appreciate it may be logistically difficult to define an exact duration, but to ensure the most robust comparison of data are the authors planning to try and perfuse for a specific duration?

Exclusion criteria of 'complex vascular anatomy' is unclear. Does this mean vascular anatomy precluding connection to the NMP circuit or a specific number of arteries?

It would be extremely valuable to have pre-implantation and post reperfusion biopsies and would it be possible to only include kidneys that have undergone a biopsy through QUOD?

VERSION 1 - AUTHOR RESPONSE

Reviewer: 1

Dr. Quan Yao Ho, Singapore General Hospital

Comments to the Author:
I thank the editors and authors for the opportunity to review this protocol.
The authors presented a protocol for a phase 1 RCT to test the addition of the CytoSorb filter to a NMP circuit for deceased donor kidney transplants.
This study will help to answer an important clinical question. The protocol is clear and well written and I have only minor comments.

1. Are there any additional treatment required for the graft while it is connected to the Cytosorb filter while on pump (e.g. additional anticoagulation)?

- *The priming solution contains 3000 units of heparin to anticoagulate the circuit. Additional treatments will be delivered on an 'as required' basis via infusion pumps connected to the circuit. The purpose of such treatments is to keep the perfusion parameters within physiological ranges. For instance, 5% glucose and bicarbonate may be delivered guided by perfusate glucose and pH measurements. Prostacyclin and verapamil may be infused to enhance renal blood flow. See page 6, Study groups.*

2. Will the hemodynamic parameters of the graft while on NMP be monitored?

- *Yes, these parameters will be continuously monitored. See page 6, Study groups.*

3. What are the device-specific adverse events that will be monitored for during the study?

- *In our randomised controlled trial of NMP in DCD kidneys transplantation there were no device specific adverse events in a series of 277 cases. Accordingly, we do not expect any device-specific adverse events, but the circuit will be monitored closely to ensure that the volume of perfusate is adequate to ensure adequate flow through the Cytosorb filter.*

4. Can inflammatory markers be measured from blood samples from the deceased donors?

- *Whilst this is out-with the scope of the primary analysis, we can in future request access to Quality in Organ Donation (QUOD) National Biobank blood samples taken from the deceased donors prior to their demise, where such samples are available and should this analysis be deemed of sufficient added value to the primary analysis.*

5. Can the authors further elaborate on the inflammatory/injury markers and markers of kidney injury that they plan to measure / are available in the panels that will be used?

- *These details have now been included. See page 9, Analysis.*

6. Can the authors provide further details on the gene expression analysis since it is a primary aim (e.g. platform and specific analysis)?

- *These details have now been included. See page 9, Analysis.*

7. Was a sample size calculation performed for the primary aim proposed?

- *As this is a pilot study, a formal sample size calculation was not performed. The sample size is pragmatic but also based on previous experimental work in human kidneys that showed that n = 5 per group was able to identify differences in transcriptional changes in human kidneys that were perfused with or with the Cytosorb filter in the perfusion circuit.*

8. It may be interesting to monitor perioperative hypotensive episodes and/or use of vasopressors

- *These will be recorded post-operatively*

Reviewer: 2

Dr. Simon Knight, University of Oxford
Comments to the Author:
This is an interesting study and a well written, clear manuscript. The primary focus is safety and feasibility, rather than efficacy.

My main suggestions relate to a lack of justification or details:

1. Inclusion criteria are fairly restrictive for a single centre study (only donors > 50, recipients on dialysis). Could the authors justify these decisions? For example, as the main focus is safety and feasibility, is there a reason to exclude pre-dialysis recipients?

- *We have excluded pre-dialysis patients because their inclusion may confound the interpretation of one of the secondary outcome measures, DGF, in our small cohort. The results of this pilot study will be used to inform the design of a randomised clinical trial with broader inclusion criteria.*

2. There is no stratification by donor type in randomisation. Whilst I suspect this is due to the small sample size, there is a risk in such a small study of an imbalance in the DBD/DCD

ratio between arms which would significantly affect the primary outcome. This should at lease be noted as a potential risk.

- *This is now acknowledged as suggested. See page 11, Limitations.*

3. Is there a maximum perfusion duration specified in the protocol?

- *The maximum perfusion duration is six hours. See page 6, Study groups.*

4. As the perfusionists will not be blinded, what steps have been taken to standardise the perfusions? A number of drugs can be infused "as required" leaving the potential for manipulation of the perfusion, for example? Does the study protocol standardise the use of such infusions (e.g. if resistance > x, give more verapamil or similar)?

- *The perfusion protocol is standardised, including the use of vasodilators.*

5. Whilst withdrawals are unlikely, will these be replaced in the randomisation schedule (e.g. if the kidney is not transplanted)? As the sample size is small, one or two withdrawals could have a significant impact.

- *Whilst consent will be sought soon after the recipient arrives on the ward, it is only after the implanting surgeon has inspected the kidney on the back bench and made a final decision to proceed with the transplant, that formal enrolment in the trial and randomisation will take place. If at the backbench there is any uncertainty as to the transplantability of the organ or the technical feasibility of machine perfusion e.g. due to arterial anomalies, then we will not proceed to formal enrolment of that recipient in the trial. The timing of enrolment therefore ensures that 'withdrawals' on account of the organ being un-transplantable are highly unlikely in this context.*
- *With regards to very remote possibility of that a recipient withdraws consent after enrolment/randomisation i.e. once machine perfusion has already taken place, the decision to replace would be revisited towards the end of recruitment period and would be contingent on the availability of funding/resources.*

6. Does the addition of the cytokine filter constitute a modification of the disposable circuit? Or are the device and circuit considered to be used "on-label" (i.e. does this need to be registered as a device trial with the MHRA)?

- *The Cytosorb filter is CE marked for clinical use in an extracorporeal circuit therefore MHRA approval is not required.*

7. The outcomes need to be defined in much more detail. What inflammatory and immune gene markers will be analysed? These should be pre-specified to avoid "fishing". What definitions of DGF and PNF will be used? Howe will transplant function be assessed? etc.

- *Definitions for DGF and PNF have now been included. See page 7, secondary outcomes.*
- *Details on the inflammatory markers and immune gene markers of interest have also been included. See page 9, Analysis.*

8. The study is described as a safety and feasibility study, yet the primary endpoint is an efficacy endpoint. How did the authors arrive at the proposed sample size? Purely a

feasible recruitment target, or is there any experimental data to demonstrate a likely difference in the primary endpoint?

- *As this is a pilot study, a formal sample size calculation was not performed. The sample size is pragmatic but also based on previous experimental work in human kidneys that showed that n = 5 per group was able to identify differences in transcriptional changes in human kidneys that were perfused with or with the Cytosorb filter in the perfusion circuit.*

9. The authors refer to QUOD in the discussion. This should be defined for a non-transplant audience. What proportion of the donors do the authors anticipate having QUOD samples available for?

- *Agreed, the definition has now been added to the discussion. See page 11, Limitations.*
- *Unfortunately, we cannot predict exactly how many donor kidneys included as part of this trial will have QUOD biopsies but estimate that this will be <50%.*

10. Figure 1 is an excellent summary of the study. The only step missing is the provision of information to patients on the WL (very minor, not essential!).

The patient information sheet will be circulated to all patients on the active Cambridge transplant list (n~230)

Reviewer: 3

Dr. Nicolette Bishop , Loughborough University
Comments to the Author:
This is a well designed study with high potential importance. The protocol has been reported in line with the SPIRIT guidelines and as such my comments are minor and only for clarification.

1) the dates for the study should be included somewhere in the manuscript, in line with the editorial requirements for protocol papers in BMJ Open.

- *We have added the study dates. See page 4, Recruitment.*

2) Analysis-page 9: While it is acknowledged that the methods will assess panels that include many different genes, cytokines and other biomarkers, it would be useful to highlight some key example expressed genes, cytokines, immune phenotypes and other biomarkers that will be assessed here, particularly for interpretation of the benefits of the future results to elucidate specific relevant mechanisms.

- *Details on key inflammatory markers and immune gene markers of interest have now been included. See page 9, Analysis.*

Reviewer: 4

Mr. James Hunter, University Hospitals Coventry and Warwickshire NHS Trust
Comments to the Author:
Thank you for the opportunity to review this interesting early phase clinical trial protocol. The study aims to randomise kidneys (n=20) to NMP with and without a

Cytosorb filter to determine the role on inflammation and inflammatory gene expression.
The aims are clear and limitations appropriately described.
minor comments

1. NMP duration of 2-6h - presumably the differences in inflammation and gene expression will vary depending upon the NMP duration. I appreciate it may be logistically difficult to define an exact duration, but to ensure the most robust comparison of data are the authors planning to try and perfuse for a specific duration?

- *We are aiming for a perfusion duration of 4 hours but have specified a range of 2-6 hours to be practical.*

2. Exclusion criteria of 'complex vascular anatomy' is unclear. Does this mean vascular anatomy precluding connection to the NMP circuit or a specific number of arteries?

- *Any arterial anatomy (including post-reconstruction arterial anatomy) precluding connection of the kidney to the NMP circuit would result in that donor organ meeting the exclusion criteria.*

3. It would be extremely valuable to have pre-implantation and post reperfusion biopsies and would it be possible to only include kidneys that have undergone a biopsy through QUOD?

- *We agree that it would be extremely valuable to have pre-implantation QUOD biopsies for comparison. However, including the absence of a QUOD biopsy as an exclusion criterion would slow down recruitment significantly, given that our current inclusion criteria are already restrictive.*
- *The local practice is to perform pre-implantation kidney biopsies for Remuzzi scoring for all donors over the age of 60, and donors over the age of 50 with significant hypertension and/or uncontrolled diabetes, and/or in cases where the donor kidney has an AKI. This should provide a useful proportion of pre-implantation biopsies.*

VERSION 2 - REVIEW

**Reviewer**	**1**
**Name**	**Ho, Quan Yao**
**Affiliation**	**Singapore General Hospital**
**Date**	**17-Feb-2025**
**COI**	

Thank you for the opportunity to review this manuscript. My previous comments have been addressed and I do not have any other concerns.

**Reviewer**	**2**

**Name**	**Knight, Simon**
**Affiliation**	**University of Oxford**
**Date**	**07-Feb-2025**
**COI**	

Thank you for the clarifications and modifications to the manuscript. It might be worth specifying the method for GFR estimation (e.g. CKD-EPI, MDRD), but otherwise the manuscript is greatly improved by additional details.

VERSION 2 - AUTHOR RESPONSE

Reviewer: 2
Dr. Simon Knight, University of Oxford
Comments to the Author:
Thank you for the clarifications and modifications to the manuscript. It might be worth specifying the method for GFR estimation (e.g. CKD-EPI, MDRD), but otherwise the manuscript is greatly improved by additional details.

**The method of eGFR caulculation has now been added to the main manuscript.

Reviewer: 1
Dr. Quan Yao Ho, Singapore General Hospital
Comments to the Author:
Thank you for the opportunity to review this manuscript. My previous comments have been addressed and I do not have any other concerns.

Please also see the revised manuscript (clean and marked copies) attached.
